# Strong Optomechanical Interaction in Hybrid Plasmonic-Photonic Crystal Nanocavities with Surface Acoustic Waves

**DOI:** 10.1038/srep13782

**Published:** 2015-09-08

**Authors:** Tzy-Rong Lin, Chiang-Hsin Lin, Jin-Chen Hsu

**Affiliations:** 1 National Taiwan Ocean University, Department of Mechanical and Mechatronic Engineering, Keelung, 20224, Taiwan; 2 National Taiwan Ocean University, Institute of Optoelectronic Sciences, Keelung, 20224, Taiwan; 3 National Yunlin University of Science and Technology, Department of Mechanical Engineering, Douliou, Yunlin, 64002, Taiwan

## Abstract

We propose dynamic modulation of a hybrid plasmonic-photonic crystal nanocavity using monochromatic coherent acoustic phonons formed by ultrahigh-frequency surface acoustic waves (SAWs) to achieve strong optomechanical interaction. The crystal nanocavity used in this study consisted of a defective photonic crystal beam coupled to a metal surface with a nanoscale air gap in between and provided hybridization of a highly confined plasmonic-photonic mode with a high quality factor and deep subwavelength mode volume. Efficient photon-phonon interaction occurs in the air gap through the SAW perturbation of the metal surface, strongly coupling the optical and acoustic frequencies. As a result, a large modulation bandwidth and optical resonance wavelength shift for the crystal nanocavity are demonstrated at telecommunication wavelengths. The proposed SAW-based modulation within the hybrid plasmonic-photonic crystal nanocavities beyond the diffraction limit provides opportunities for various applications in enhanced sound-light interaction and fast coherent acoustic control of optomechanical devices.

The use of ultrahigh-frequency acoustic waves to manipulate or modulate the optical behaviors of micro- and nanostructures has recently drawn significant attention in the fields of nano-scale photonics and fundamental optical physics^1^,^2^,^3^,^4^. Linear light modulation by acoustic phonons in the interior of homogeneous bulk materials relies on the influence of local stress field induced by the acoustic waves, which is known as the photoelastic effect^5^,^6^. Furthermore, the light modulation can potentially be enhanced through the disturbance of the structural interfaces by acoustic waves, which is termed the interface effect^7^,^8^,^9^. On the other hand, carefully engineered optical modes, such as waveguide, cavity, or slow-light modes in nanostructured materials are often used to achieve enhanced nonlinear interactions between photons and phonons^10^,^11^,^12^. Recently, considerable enhancement of photon-phonon and optomechanical interactions has involved the utilization of dual photonic-phononic bandgap structures and micro- or nanocavities with optical modes efficient in the multiphonon absorption and emission, in which high densities of photon and phonon states can be simultaneously be tightly confined to the same small region of space^13^,^14^,^15^,^16^. In an optical nanocavity, the (cold-cavity) quality factor *Q* is used to describe the spectral energy density of the cavity modes and how long the stored energy of the cavity mode remains in the cavity. Because the *Q* factor is intrinsically proportional to the cavity photon lifetime, nanocavities with high *Q* have been exploited to achieve efficient optomechanical interaction together with the photoelastic and interface effects. Recently, nanocavities have been designed to simultaneously provide high *Q* factors for both photon and phonon resonances to trigger multiphonon processes that enable much stronger photon-phonon interactions^17^,^18^,^19^. Strong optomechanical interaction may lead to several fundamental studies of quantum effect and optical physics in nanosystems, such as optical cooling of mechanical oscillators in the quantum regime, enhancement of optical nonlinearity, and development of optomechanical crystals and devices^20^,^21^,^22^,^23^,^24^,^25^,^26^. Although conventional dielectric nanocavities can be designed to have very high *Q* factors^27^, the phonon disturbance is still restrictive in that acoustic intensity cannot be amplified freely because of limited excitation efficiency or because the mechanical strain must be maintained well below the elastic limit of the material^28^,^29^. A possible alternative for further enhancing optomechanical interaction is to design the optical wavelength beyond the diffraction limit so that the achievable or allowable acoustic amplitude is comparable to the characteristic scale of the optical cavities.

The optical cavity mode can be designed beyond the diffraction limit by forming the surface plasmons at the metal-dielectric interface^30^,^31^. Surface plasmons are highly potential to generate intense optical fields in an ultra-compact cavity to accelerate light-matter interaction by boosting the so-called Purcell factor (proportional to the ratio of *Q* factor and mode volume, *Q*/*V*_m_), which has been used in extremely sensitive nano-scale spectroscopy^32^,^33^. Surface plasmon-assisted lasing was demonstrated in several previous reports on topics such as gold nanospheres coated with a gain medium^34^, metal-cladding nanoresonator^35^,^36^, insulator-metal nanowaveguide^37^,^38^,^39^, and plasmonic band gap nanocavity^40^,^41^,^42^. Plasmonic nanocavities can achieve deep subwavelength mode volume; however, the *Q* factor are limited by the significant material loss of metal.

Recently, hybrid optical nanocavities have been proposed to compress optical energy into deep subwavelength regions^37^,^43^,^44^. The systems hybridize the photonic and surface plasmonic modes to form surface plasmon polaritons (SPPs), resulting in tighter spatial confinement of optical energy, higher local field intensity, and lower parasitic loss of metal^45^,^46^. These properties can be used to enhance the efficiency of the optomechanical interaction between the photonic and phononic modes. In this study, we demonstrate the strong modulation of SPP modes at telecommunication wavelengths by surface acoustic waves (SAWs). The SPP modes were strictly confined to a nanocavity which consisted of a defect-containing photonic crystal beam and a metal surface with a nanoscale air gap in between, as shown schematically in Fig. 1a. The one-dimensional periodic beam contained defects in the middle by removing or modifying the air holes and was set up above a metal substrate with an air gap in between. The structure could then be designed to have both a high-*Q* factor and deep subwavelength mode volume *V*_m_ because the hybrid system supported a strong optical energy confined in the low-loss air gap region. Though the hybrid structure also introduced several photonic loss mechanisms, such as in-plane SPP radiation, evanescent coupling with the dielectric beam, and metal absorption^47^, it achieved a much higher *Q*/*V*_m_ ratio (or smaller mode volume *V*_m_), which relates more directly than does the *Q* factor to the enhancement of photon-phonon interactions that allow for intense high-frequency acoustic disturbance. In this report, we will demonstrate the enhanced optomechanical modulation of the SPP mode in a nanocavity of high *Q* and low *V*_m_ using monochromatic coherent acoustic phonons localized on the silver substrate surface formed by high-frequency SAWs. In addition, we will systematically study the influential factors in such a system.

## Results and Discussion

**Figure 1:**
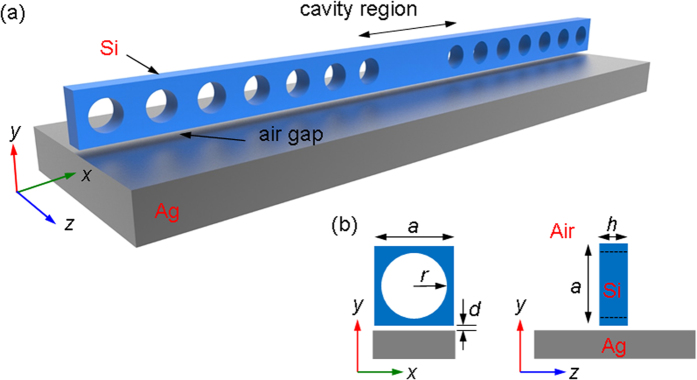
(**a**) Schematic of the hybrid plasmonic-photonic crystal nanocavity consisting of two missing circular holes in the cavity region. A nanobeam is set up above a metal (silver) substrate with an air-gap separation of distance *d*. (**b**) Geometry of the unit cell of the one-dimensional hybrid plasmonic-photonic crystal. The nanobeam is assumed to be made of silicon. The lattice constant *a* **=** 450 nm, the hole radius *r* **=** 135 nm (=0.3*a*), and the beam thickness *h* **=** 200 nm.

**Figure 2:**
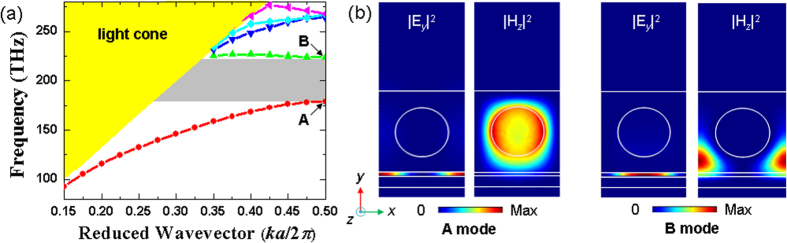
(**a**) Band structure of the hybrid plasmonic-photonic crystal without defect, where *d* **=** 20 nm. The shaded region represents the light cone. A band gap exists from 179–224 THz. The lower and upper band-edge modes at the BZ boundary are denoted as *A* and *B* modes, respectively. (**b**) The corresponding distributions of electric field |*E*_*y*_|^2^ and magnetic field |*H*_*z*_|^2^ of *A* and *B* modes.

Figure 1b shows the geometry of a unit cell of the crystal nanostructure composed of a photonic crystal beam. The structure consisted of silicon with periodic circular air holes along the *x*-axis and was separated from a silver substrate by a nano air gap of distance *d*. The lattice constant was *a* **=** 450 nm, the hole radius was *r* **=** 135 nm (=0.3*a*), and the beam thickness was *h* **=** 200 nm. Figure 2a shows the band structure of the non-defect crystal nanostructure, in which a band gap (marked by the gray region) below the light cone is found from 179–224 THz (with corresponding wavelength is from 1339–1676 nm). We chose the air gap separation to be *d* **=** 20 nm. The magnetic field **H** and electric field **E** exhibited TM polarizations with plasmonic-photonic hybridization for both the lower and upper bands of the band gap. We denote the lower band-edge mode (at 179 THz) and upper band-edge mode (at 224 THz) at the Brillouin zone (BZ) boundary as modes *A* and *B*, respectively, and plot their electric field |*E*_*y*_|^2^ and magnetic field |*H*_*z*_|^2^ distributions in Fig. 2b. The electric fields of modes *A* and *B* were strongly concentrated in the low-loss air gap because of the larger coupling between the silicon photonic mode and the surface plasmonic mode. Hybrid modes *A* and *B* form distinct distributions with their electric field concentrated in the gap underneath the holed and unholed regions, respectively, resulting in separation of a continuous SPP band and formation of the band gap at the BZ boundary.

**Figure 3:**
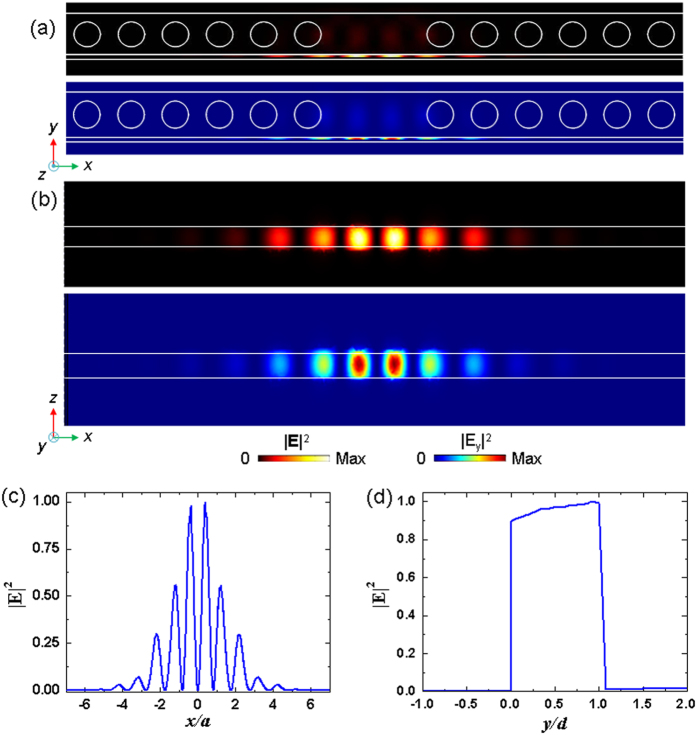
|E|^2^ and |*E*_*y*_|^2^ field distributions of the SPP cavity mode on the (a) *x-y* and (b) *x-z* planes, respectively. The result is obtained using the supercell approach with seven lattice periods on each side of the cavity. The cavity mode is confined by the cavity and squeezed inside the air gap. Variations of the |**E**|^2^ field for the cavity mode (**c**) at the center of the air gap along the *x*-direction and (**d**) close to the middle of the defect along the *y*-direction, respectively. In (**c**) *x*/*a* **=** 0 denotes the cavity center, and in (**d**) *y*/*d* **=** 0 denotes the silver substrate surface.

We generated defects in the structure to serve as a nanocavity by removing two circular air holes at the middle of the silicon photonic crystal beam, as shown in Figs 1a and 3a. The nanocavity supported a highly confined SPP cavity mode by means of the simultaneous SPP and bandgap effects, which confine propagation along the *z-*direction and *x*-direction, respectively. Fig. 3a,b show clearly the confinement of the field distributions |**E**|^2^ and |*E*_*y*_|^2^ on the *x-y* and *x-z* planes, respectively. The electric field intensity |**E**|^2^ was strongly squeezed within the 20-nm air gap; therefore, the optical resonance may have been sensitive to the perturbation due to this deep subwavelength air gap of width *d*. The eigenfrequency of the SPP cavity mode was 193.6 THz with a corresponding resonance wavelength of *λ*_*r*_ **=** 1548.71 nm. The mode volume and quality factor were *V*_m_ **=** 14 × 10^−3^*λ*_*r*_^3^ and *Q* **=** 544, respectively. Fig. 3c,d show the variations of the |**E**|^2^ field at the center of the air gap along the *x*-direction and close to the middle of the defect along the *y*-direction, respectively. The |**E**|^2^ field distribution is symmetric with respect to the middle of the cavity along the *x*-direction and rapidly decays away from the air gap region.

**Figure 4:**
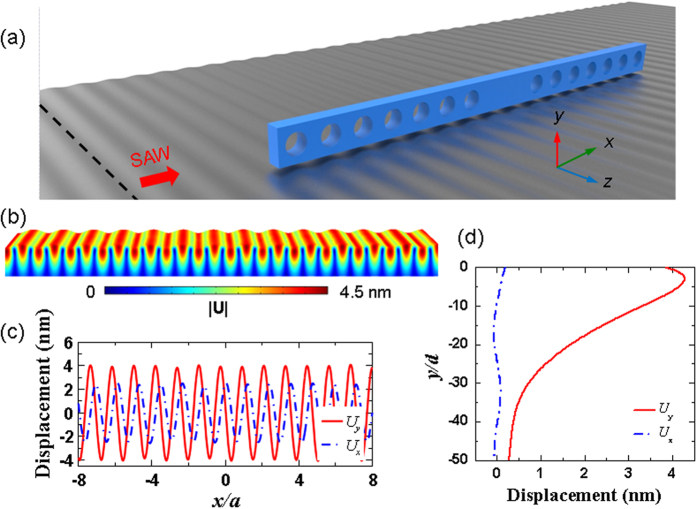
(**a**) Schematic of the perturbation of the crystal nanocavity using 3-GHz Rayleigh SAWs propagating along the *x-*direction on the surface of the silver substrate. (**b**) Calculated SAW field distribution on the surface of the silver substrate with a total displacement amplitude |**U**| equal to 4.5 nm. (**c**) The corresponding displacement components *U*_*y*_ and *U*_*x*_ of the SAW field, where the amplitude of *U*_*y*_ **=** 4.0 nm. (**d**) Variation of the displacements of Rayleigh SAWs along the depth from the silver substrate surface.

On the basis of the high-*Q* and low-*V*_m_ plasmonic-photonic nanocavity, we then consider the optomechanical effect induced by high-frequency Rayleigh SAWs propagating along the *x*-direction on the surface of the silver substrate, as shown schematically in Fig. 4a. In reality, one can employ piezoelectric material with comb-shape electrodes to electrically generate high-frequency SAW on the silver surface^48^,^49^,^50^. For example, depositing piezoelectric thin film (e.g. ZnO) on the silver substrate or using crystalline piezoelectric substrate (e.g. LiNbO_3_) to support a silver layer, instead of using bulk silver substrate. The Rayleigh SAWs were elliptically polarized on the *x-y* plane, which effectively perturbed the SPP mode concentrated in the air gap to induce strong photon-phonon interaction. Figures 4b–d show the total displacement field |**U**|, vertical and horizontal displacement field components *U*_*y*_ and *U*_*x*_ on the silver substrate surface, and depth-dependent displacements of Rayleigh SAWs with a frequency *f*_SAW_ of 3 GHz (where the depth was measured into the silver substrate). The SAW field could be generated as traveling or standing waves localized on the silver substrate surface and induce a periodic corrugated surface of period equal to the SAW wavelength *λ*_SAW_ to perturb the crystal nanocavity. The SAW perturbation gave rise to Bragg scattering of the electromagnetic field and optomechanical interaction. In this nanocavity scheme, the interface effect dominated the optomechanical interaction. In the present work, we focus on the interaction between SAW field and SPP cavity mode. However, mechanical oscillation in the photonic crystal beam may also contribute to the optomechanical interaction through the mechanism that SAW field perturbs the stored optical power in the nanocavity to have an optical power variation related to the SAW frequency and regenerate the mechanical oscillation of the photonic crystal beam^51^,^52^,^53^. As a result, the interaction between the SPP cavity mode and mechanical oscillation may be enhanced.

**Figure 5:**
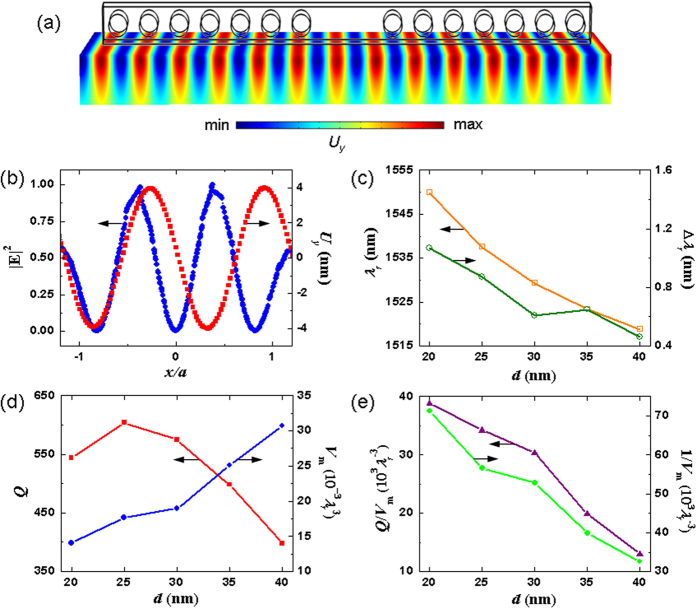
(**a**) An arbitrarily chosen SAW phase relative to the nanocavity. (**b**) Comparison of spatial distributions between the traveling SAW field at the chosen phase and the SPP cavity mode. (**c**) Resonance wavelength *λ*_*r*_with different air gap width *d* and the corresponding shift Δ*λ*_*r*_ of the SPP cavity mode perturbed by the 3-GHz SAWs with a restricted amplitude *U*_*y*_ of 4 nm. The dependences of (**d**) the *Q* factor, mode volume *V*_m_, (**e**) 1/*V*_m_, and *Q*/*V*_m_ ratio for the SPP cavity mode.

**Figure 6:**
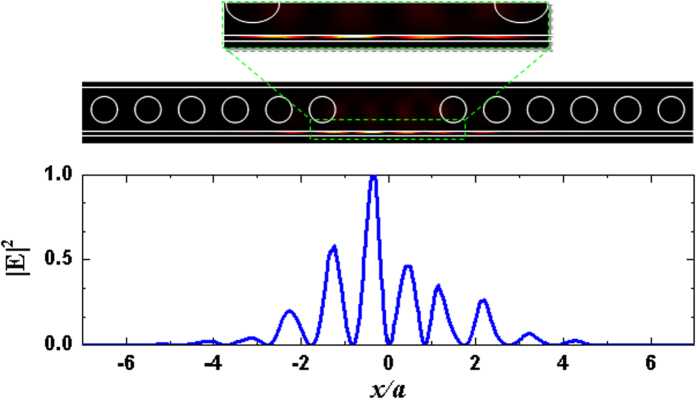
|E|^2^ field distribution of the SPP cavity mode with *d* = 20 nm perturbed by the 3-GHz traveling SAWs at the chosen phase shown in Fig. 5a. The perturbed |E|^2^ field has a redistributed asymmetric concentration pattern. The resulting shift is Δ*λ*_*r*_ **=** 1.07 nm with a resonance wavelength *λ*_*r*_ = 1549.78 nm.

To relate the resonance wavelength shift Δ*λ*_*r*_ of the SPP cavity mode perturbed by the SAW field to the resonant mode characteristics, their trends as a function of the air-gap width *d* were compared. The SAW field lay in an arbitrarily chosen phase relative to the unperturbed SPP cavity mode, as shown in Fig. 5a,b. Figure 5c shows the resonance wavelength *λ*_*r*_ of the SPP cavity mode with different air gap width *d* and the corresponding shift Δ*λ*_*r*_ which was perturbed by the 3-GHz SAWs with a restricted amplitude of *U*_*y*_ **=** 4 nm. When the air gap *d* was increased, the resonance wavelength decreased because the coupling of the silicon photonic mode and surface plasmonic mode was reduced with a decreased effective index^47^,^54^, and the wavelength shift was smaller because the interface effect at larger *d* values becomes weaker for photon-phonon interaction. The dependences of the *Q* factor, mode volume *V*_m_, inverse of mode volume 1/*V*_m_, and *Q*/*V*_m_ ratio on gap width *d* for the SPP cavity mode are shown in Fig. 5d,e. Figure 5e exhibits a positive correlation between the 1/*V*_m_ and *Q*/*V*_m_ ratio and wavelength shift Δ*λ*_*r*_ from Fig. 5c, while the influence of the strength of the interface effect was dominated by the low optical mode volume because small *V*_m_ is susceptible to the deformation. The SAW field also broke the symmetry of the cavity geometry, resulting in an asymmetric SPP cavity mode shape. Figure 6 illustrate the |**E**|^2^ field distribution of the SPP cavity mode with *d* **=** 20 nm perturbed by the SAW field. The perturbed |**E**|^2^ field has a redistributed concentration pattern, corresponding to a wavelength shift Δ*λ*_*r*_ of 1.07 nm (*λ*_*r*_ = 1549.78 nm).

**Figure 7:**
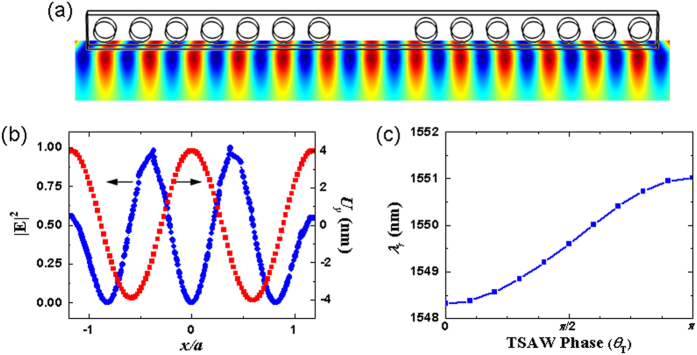
(**a**) The instant of time at the highest spatial correlation between the 3-GHz TSAWs at *θ*_*T*_ **=** *π* and the SPP cavity mode. (**b**) The corresponding |**E**|^2^-field and *U*_*y*_-field distributions in the cavity. (**c**) Evolution of the resonance wavelength *λ*_*r*_ by changing the TSAW phase *θ*_*T*_ **=** 2π*f*_SAW_*t*.

To understand the dynamic response of the SAW-base optomechanical interaction in the crystal nanocavity, we analyzed the resonance wavelength *λ*_*r*_ with different SAW properties. SAWs can be excited as traveling waves (TSAWs) or constructed to form standing waves (SSAWs) using, for example, an acoustic cavity or two-beam interference on a surface. Because the SAW frequency is five orders of magnitude smaller than that of the optical mode of comparable wavelength, the SAW field perturbation to the optical nanocavity are regarded as quasi-static. As a result, at any instant of time, the SSAW scheme can be viewed as the TSAW scheme at a specific phase, but with a varying amplitude associated with the SSAW phase *θ*_*S*_. First, we considered the highest spatial correlation of the 3-GHz TSAWs to be at another phase defined as *θ*_*T*_ **=** *π* with the SPP cavity mode, as shown in Fig. 7a,b. The resonance wavelength shift was increased to Δ*λ*_*r*_ **=** 2.31 nm. Figure 7c shows the evolution of the resonance wavelength *λ*_*r*_ by changing the TSAW phase *θ*_*T*_ **=** 2π*f*_SAW_*t*, in which the resonance wavelength monotonically increases with the change of the SAW phase *θ*_*T*_ from 0 to π. The total bandwidth Δ*λ*_*c*_ in the modulation of the resonance wavelength with the same period as the 3-GHz TSAWs increased to 2.7 nm. As a result of the optical energy being squeezed inside the deep subwavelength region of space, optical modulation by the acoustic perturbation on the interface was effective.

**Figure 8:**
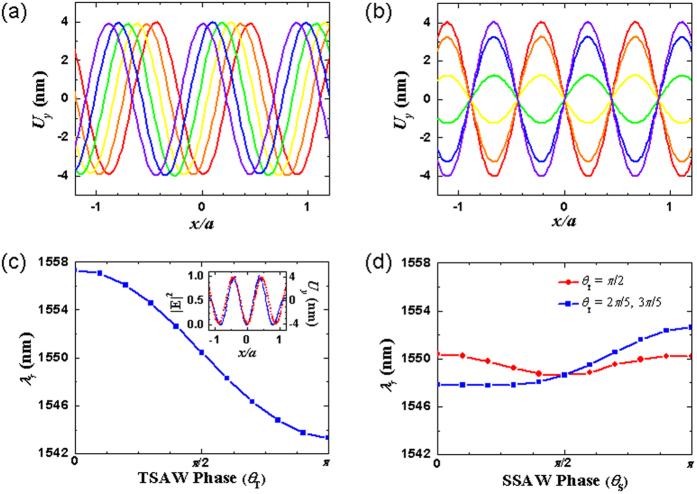
(**a**) TSAW and (**b**) SSAW schemes with *f*_SAW_ **=** 4 GHz at different phases, where the TSAWs propagate their energy forward, and the SSAWs remain in constant positions with no net propagation of acoustic energy. (**c**) Evolution of the resonance wavelength *λ*_*r*_ by changing the 4-GHz TSAW phase *θ*_*T*_. The inset shows the status *θ*_*T*_ **=** 0. (**d**) Evolution of the resonance wavelength *λ*_*r*_ perturbed by 4-GHz SSAW fields with different nodal points (corresponding to the instant of time at *θ*_*T*_ **=** π/2 and *θ*_*T*_ **=** 3π/5 and 2π/5 that determine the upper and lower bounds of the changes of *λ*_*r*_) as a function of SSAW phase *θ*_*S*_.

The strength of the optomechanical interaction also highly correlated to the wavelength of the SAW field. We examined the nanocavity modulation at several SAW frequencies (2, 3, 4, and 5 GHz) corresponding to different SAW wavelengths (*λ*_SAW_ **=** 1.78*a*, 1.15*a*, 0.91*a*, and 0.67*a*, respectively). Table 1 lists the unperturbed resonance wavelength *λ*_*r*_ and Δ*λ*_*r*_ and Δ*λ*_*c*_ of the SPP cavity mode under the perturbation of the SAW fields with different frequencies and phases based on the TSAW scheme. The SAW field of 4-GHz frequency exhibited the strongest modulation for the optical resonance wavelength shift and total bandwidth, which correspond to Δ*λ*_*r*_ **=** 8.58 nm and Δ*λ*_*c*_ **=** 13.95 nm, respectively. The wavelength of the 4-GHz SAW field closely matches the spacing of the two closest nodal points of the |**E**|^2^ field profile of the SPP cavity mode, demonstrating the condition (wavelength matching) which maximizes the interface effect for the photon-phonon interaction. The modulation using the SSAW scheme can be derived from the results of TSAW scheme. Figure 8a,b compare the traveling and standing SAW schemes, respectively. The TSAWs propagate their acoustic energy forward, whereas SSAWs remained in a constant position with no net propagation of acoustic energy. Figure 8c illustrates the resonance wavelength variations of the SPP cavity mode perturbed by the 4-GHz SSAW field with different phases (Fig. 8a) related to that perturbed by a TSAW field of the same frequency and maximum amplitude. The inset of Fig. 8c shows the spatial relation between the TSAW and SPP cavity mode. Figure 8d corresponds to the SSAW fields with nodal points located at the middle of the cavity and 0.3*λ*_SAW_ from it, respectively. Then these two SSAW fields can be regarded as identical to the TSAW fields at the instant of time at which the phase are *θ*_*T*_ **=** π/2 and *θ*_*T*_ **=** 3π/5 and 2π/5, respectively. As a result, the upper and lower bounds for the range of *λ*_*r*_ variation caused by a SSAW field can be determined using Fig. 8c with the corresponding values of *θ*_*T*_.

**Figure 9:**
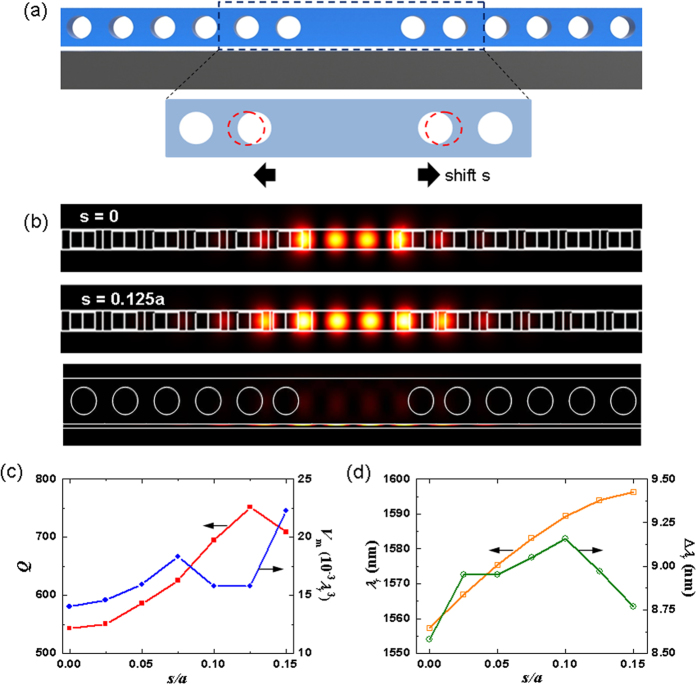
(**a**) Schematic of optimization for the crystal nanocavity by shifting the two neighbor circular holes of the defect region outward from their lattice point by a distance *s*. (**b**) The electric field intensity |**E**|^2^ distributions with *s* **=** 0 and 0.125*a*, where the mode mismatch with *s* **=** 0.125*a* is reduced to achieve a higher *Q* factor. (**c**) Variations of *Q* and *V*_m_ as a function of the shift distance *s*. (**d**) Variations of *λ*_*r*_ and Δ*λ*_*r*_ with the increasing of *s* under the perturbation of 4-GHz TSAW field of amplitude *U*_*y*_ **=** 4 nm at *θ*_*T*_ **=** 0, where the maximum Δ*λ*_*r*_ occurs at *s* **=** 0.1*a*.

To achieve a stronger optomechanical effect with a more sophisticated optical nanocavity, we optimized the crystal nanocavity by shifting the two neighbor circular air holes of the defect region outward from their lattice point by a distance *s*, as shown in Fig. 9a. Compared with the case of *s* **=** 0, Fig. 9b shows that the electric field intensity |**E**|^2^ with *s* **=** 0.125*a* has reduced mode mismatch with a larger cavity length so that the *Q* factor is increased. Figures 9c shows the variations in the quality factor *Q* and mode volume *V*_m_ as functions of the shift distance *s*. Increasing the distance *s* from 0 to 0.125*a* increased the resonance wavelength and *Q* factor, while the mode volume *V*_m_ was not significantly influenced. The highest *Q*/*V*_m_ ratio occurred at *s* **=** 0.125*a*. The variations of *λ*_*r*_ and Δ*λ*_*r*_ with increasing *s* under the perturbation of the 4-GHz SAW field of amplitude *U*_*y*_ **=** 4 nm with *θ*_*T*_ **=** 0 are shown in Fig. 9d. The refined effective cavity length further improves the SPP confinement to increase the wavelength shift Δ*λ*_*r*_ by the SAW field. With *s* **=** 0.1*a*, the wavelength shift Δ*λ*_*r*_ achieved 9.16 nm. *Q* and *V*_m_ can be further increased and reduced, respectively, to boost the optomechanical interaction or photon-phonon interaction by optimizing the overall geometry of the SAW-based tuning nanocavity structure, when optical losses and air gap width are optimized. The ultrahigh-frequency SAW field can also be tailored to provide a resonant phonon intensity distribution that closely resembles or correlates to the spatial mode profile of the hybrid optical field using phononic bandgap structures to enhance the multiphonon absorption and emission by a photon and to increase the photon-phonon interaction time^55^,^56^,^57^.

## Conclusion

We have studied the optomechanical effect of high-frequency SAW-based modulation on a crystal nanocavity. The enhancement of the photon-phononic interactions was achieved through an efficient interface effect using the SAW disturbance at the nanoscale air gap of the nanocavity where the deep subwavelength hybrid optical energy is highly concentrated. A large modulation bandwidth Δ*λ*_*c*_ of 13.95 nm and resonance wavelength shift Δ*λ*_*r*_ of 8.58 nm with a 4-GHz SAW field were demonstrated at telecommunication wavelength for the high *Q* and low *V*_m_ crystal nanocavity. We correlated the influences of the *Q* factor and mode volume *V*_m_ of the crystal nanocavity on the optomechanical interaction with the perturbation of the high frequency SAWs. Additionally, we demonstrated the feasibility of achieving efficient optomechanical interaction beyond the optical diffraction limit. This study can be used to realize many applications involving enhanced sound-light interaction, such as nanolaser cooling, light modulation, quantum motion, and phonon laser action.

## Method

### Simulation

The numerical calculations of the optical and acoustic wave field were performed using the finite-element method (FEM) software package (COMSOL Multiphysics) with the RF and Structural Mechanics modules combined and a moving mesh tool for field interaction. In the optical modeling, the used refractive indices were *n*_Si_ **=** 3.46 for silicon and *n*_Ag_ **=** *n*_R_ + *jn*_I_ for silver based on the experimental data that considers the dispersive property^58^. For the electromagnetic eigenmode and eigenfrequency solutions, the Bloch periodic boundary conditions (PBCs) as a function of wavevector **k** were applied along the propagation direction (i.e., the *x*-direction) on the boundaries of the unit-cell model, and continuous PBCs are applied on other boundaries. For the solutions of the cavity modes, the supercell model that contains 16 lattices and two missing air circular holes in the middle of the photonic crystal beam was adopted with PBCs. The mode volume of the SPP cavity mode is calculated using^43^,^59^


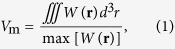


where *W*(**r**) is the optical energy density at position **r**, given by


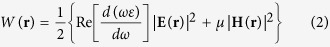


with *ω* being the optical frequency, and *ε* and *μ* being the associated permittivity and permeability, respectively. The quality factors of the cavity mode were obtained using the formula *Q* **=** *λ*_*r*_*/*Δ*λ*, with Δ*λ* being the full width at half-maximum of the resonance intensity spectrum, where the spectrum was calculated using the FEM-based frequency response analysis with *y*-polarized electric dipole excitation located in the cavity. The well-known perfectly matched layers (PMLs) were employed at the calculated domain boundaries to eliminate the reflection of outgoing electromagnetic waves. The SAW wavelength was estimated by solving the equation for the Rayleigh SAW velocity *c*_R_ in elastic isotropic media^60^


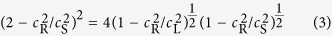


with *c*_L_ and *c*_S_ being the longitudinal and transverse bulk acoustic wave velocities, respectively, and the relation *c*_R_ **=** *f*_SAW_*λ*_SAW_. In the FEM modeling of the acoustic field, the SAW displacement field 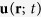 was calculated based on the elastodynamic wave equation^61^


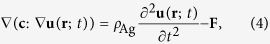


with a mass density *ρ*_Ag_ **=** 10,500 kg/m^3^ and elastic stiffness tensor **c** which is expressed in terms of Young’s modulus *E* **=** 83 GPa and Poisson’s ratio *ν* **=** 0.37 for the silver substrate. **F** is the forcing term for the excitation of the SAW field setting on the silver substrate surface. A sinusoidal line force source was applied in the simulations, and acoustic PMLs were used at the calculated domain boundaries to eliminate the reflection of outgoing SAWs and radiated bulk acoustic waves. In analyzing the SAW field, we neglected the acoustic attenuation because the crystal nanocavity covers only several SAW wavelengths. Typical attenuation coefficient *α* in the gigahertz regime is proportional to square of acoustic frequency. Estimation with an attenuation coefficient *α* **=** 10^4^ dB/m^5^, the attenuation of the SAW amplitude was minor during such short-distance propagation. Then the SAW disturbed optical model was built through the moving mesh technique in the FEM calculations for the interaction of electromagnetic and acoustic fields.

## Additional Information

**How to cite this article**: Lin, T.-R. *et al.* Strong Optomechanical Interaction in Hybrid Plasmonic-Photonic Crystal Nanocavities with Surface Acoustic Waves. *Sci. Rep.* **5**, 13782; doi: 10.1038/srep13782 (2015).

## Figures and Tables

**Table 1:** Modulation of the SPP cavity mode using TSAWs of different frequencies and phases.

*f*_SAW_ (GHz)	*λ*_SAW_	|*U*_*y*_|(nm)	*λ*_*r*_(nm)	Δ*λ*_*r*_(nm)			Δ*λ*_*c*_(nm)
				*θ*_*T*_ = 0	*θ*_*T*_ = *π*/2	*θ*_*T*_ = *π*	
2	1.78*a*	4.0	1548.71	1.66	0.64	−0.34	2.0
3	1.15*a*			–0.39	0.89	2.31	2.7
4	0.91*a*			8.58	1.73	−5.37	13.95
5	0.67*a*			4.0	1.45	−0.92	4.92
